# The Science and Art of Surgery

**Published:** 1861-05

**Authors:** 


ART. X.—*The Science and Art of Surgery, being a Treatise on Surgical Injuries, Diseases, and Operations.* By JOHN E. ERICHSEN. Third edition, enlarged and carefully revised. London, 1861.

HARDLY eight years have elapsed since the first appearance of this work. The author has, therefore, just cause to be proud of the favor which it has received from the profession at home and abroad. The present edition is the third, which was issued at London, in November, 1860.

"Every page," says Mr. Erichsen, "has been carefully revised. The text has been considerably enlarged, and in many parts rearranged. Several new chapters have been added, and many new illustrations introduced. The table of contents and the index have been rendered as complete as possible, so as to facilitate reference to the numerous subjects treated of in the body of the work."

Four hundred and fifty engravings accompany the present edition, which may, therefore, in reference both to these and other matters, be regarded as a great improvement upon the second edition, issued in this city in the latter part of 1859. One of the most interesting additional chapters in the new edition is that on "sacro-iliac disease," a strumous affection of the sacro-iliac articulation, very rare in this country, but sufficiently frequent in Great Britain, where Mr. Erichsen was the first to give a clear and comprehensive account of it. We regret that our limits do not permit us to transfer some of his valuable remarks upon this disease to our pages.

It would be superfluous to speak of a work the merits of which are so universally acknowledged as those of Mr. Erichsen's Science and Art of Surgery. The treatise is a model of its kind, and our only regret is that the distinguished author has not bestowed the same amount of attention upon the diseases of the eye and ear that he has devoted to other subjects. This omission necessarily leaves the work imperfect, and compels the student to supply himself with separate treatises on ophthalmic and aural surgery.

The present edition is comprised in one thick octavo volume of 1167 pages, closely printed in small type, and affords, therefore, an immense amount of reading matter. The wood-cuts are well executed, and the whole mechanical performance reflects great credit upon the publishers, Messrs. Walton & Maberly.

